# Supplementary material for: The involvement of the gut microbiota in postoperative cognitive dysfunction based on integrated metagenomic and metabolomics analysis
Source: Microbiol Spectr. 2023 Nov 21;11(6):e03104-23. doi: 10.1128/spectrum.03104-23 (PMC10714990; doi:10.1128/spectrum.03104-23)
Supplement: Supplemental material — Fig. S1 to S3. [file spectrum.03104-23-s0001.pdf]

The involvement of the gut microbiota in postoperative cognitive dysfunction based on integrated metagenomic and metabolomics analysis

Shi-hua Zhang[1,2#], Xiao-yu Jia[1#], Qing Wu[1,2], Jia Jin[2], Long-sheng Xu[1], lei Yang[1], Jun-gang Han[1], Qing-he Zhou[1]

[1]Department of Anaesthesiology and Pain Medicine, the Affiliated Hospital of Jiaxing University, Jiaxing, Zhejiang Province, 314001 China

[2]College of Life Science and Medicine, Zhejiang Sci-Tech University, Hangzhou, Zhejiang Province, 310018 China

#Shi-hua Zhang and Xiao-yu Jia contributed equally to this work as co-first authors

*Correspondence Author:

Qing-he Zhou, Ph.D., Department of Anaesthesiology and Pain Medicine, the Affiliated Hospital of Jiaxing University, No.1882, South Central Road, Jiaxing 314000, China;

Tel: +86-0573-89997760

Fax: +86-0573-89997760

Email: zqh10980@zjxu.edu.cn

Jun-gang Han, Ph.D., Department of Anaesthesiology and Pain Medicine, the Affiliated hospital of Jiaxing University, No.1882, South Central Road, Jiaxing 314000, China;

Tel +86-0573-89997760

Fax +86-0573-89997760

Email: jghan@163.com

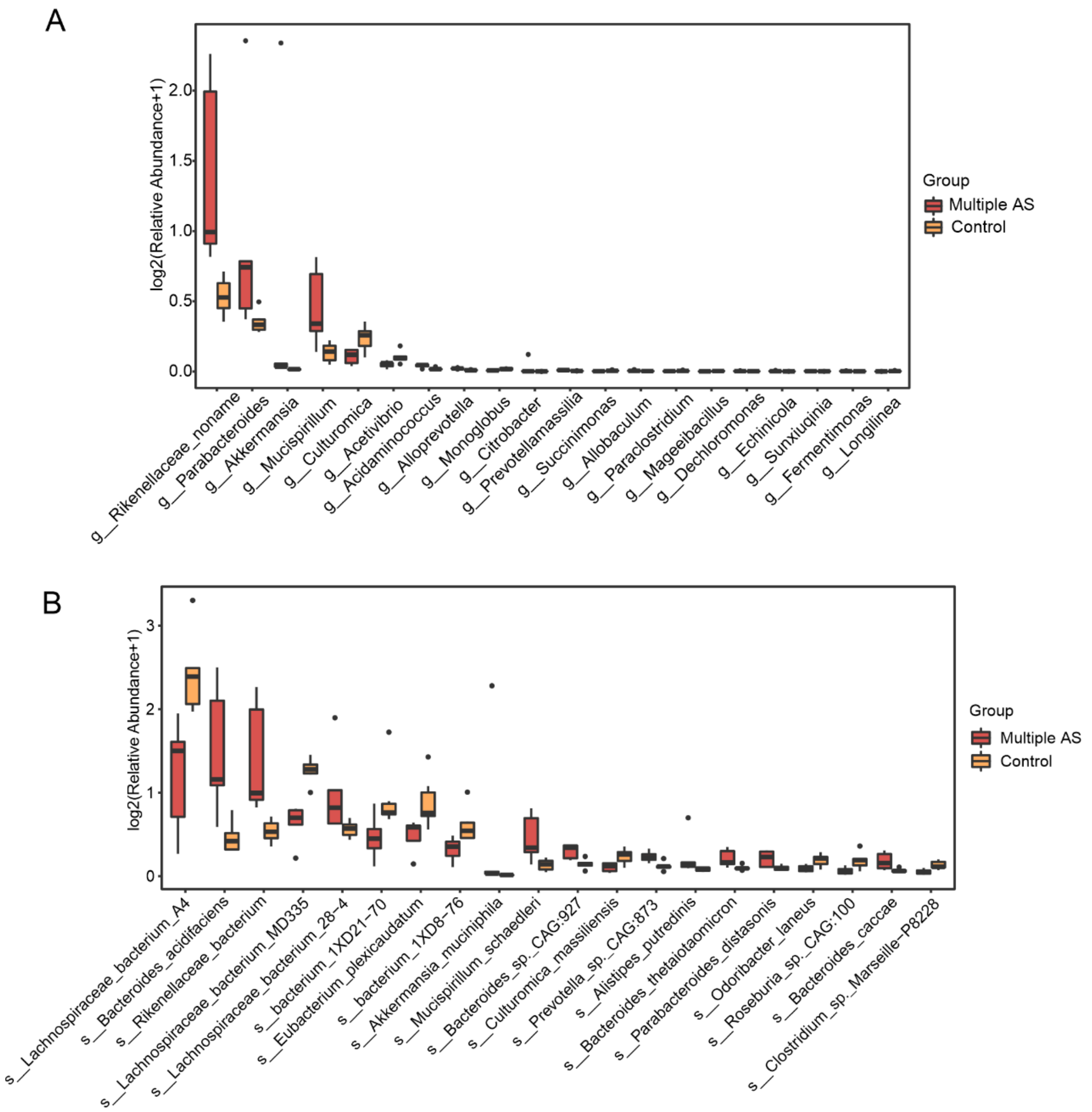

Figure S1 Statistical results regarding the relative abundance of varying flora. **(A)** Statistical box plots of relative abundance at genus level. **(B)** Box plots of species-level relative abundance statistics. P<0.05 was considered statistically significant.

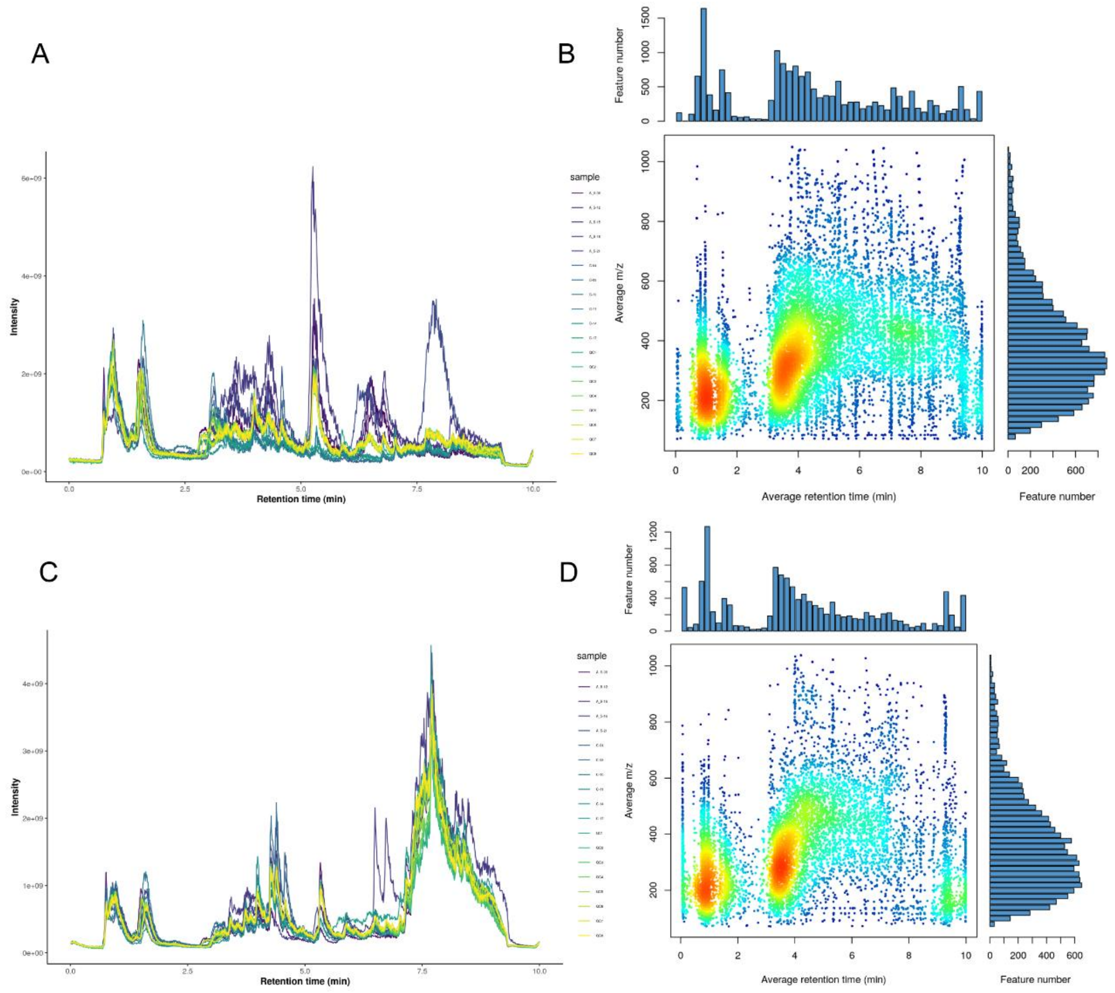

Figure S2 The total ion current diagram (TIC) shows the separation of all metabolites in liquid chromatography at each time point. **(A)** Total ion diagram in positive ion mode. **(B)** The distribution map of metabolites in positive ion mode after peak comparison of mass spectrum data by XCMS software. **(C)** Total ion diagram in negative ion mode. **(D)** The distribution map of metabolites in negative ion mode after peak comparison of mass spectrum data by XCMS software.

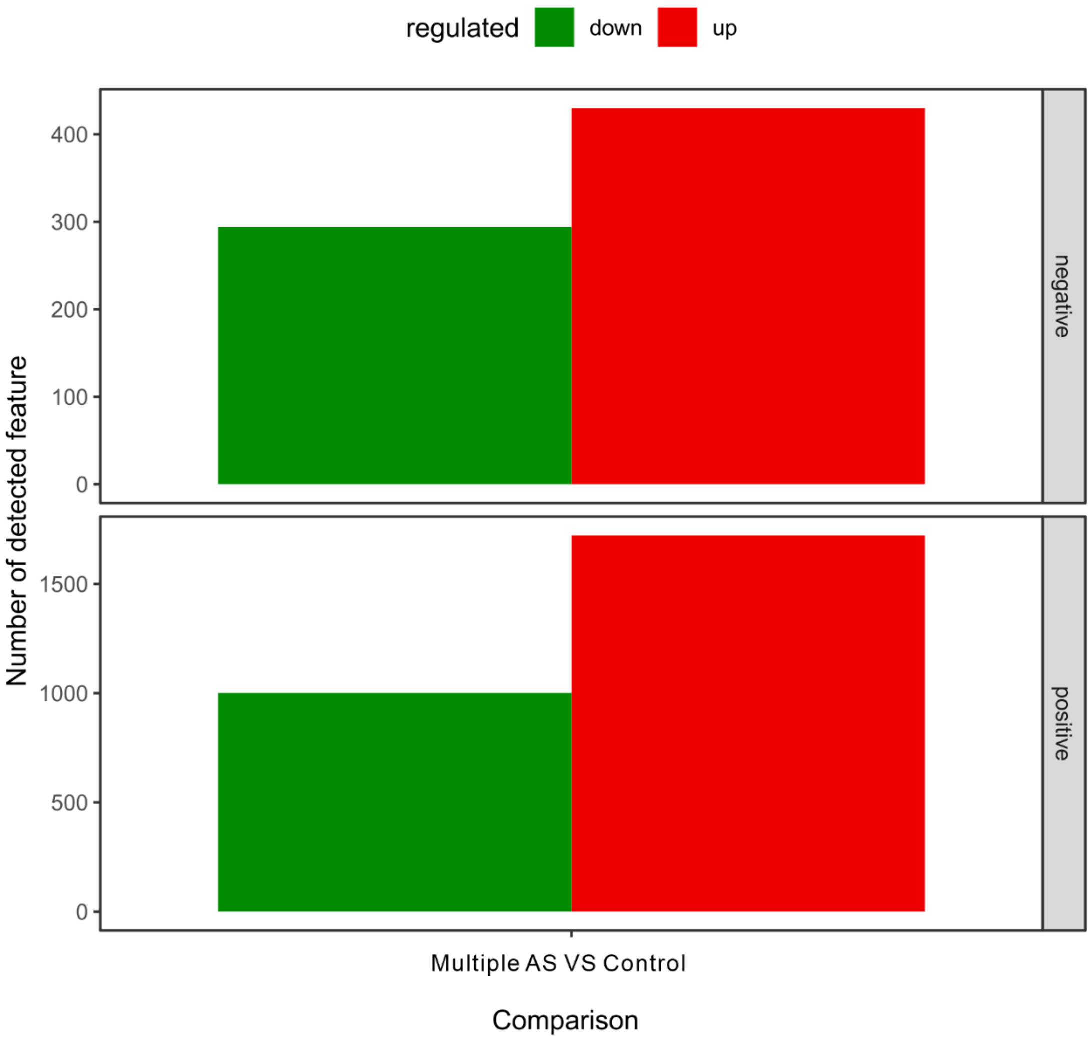

Figure S3 Differential ions in positive and negative ion modes
